# A 16-YEAR FOLLOW-UP OF WALKING FUNCTION, FATIGUE, AND PAIN IN ADULTS AGED 34–65 YEARS WITH SPASTIC CEREBRAL PALSY

**DOI:** 10.2340/jrm.v57.43295

**Published:** 2025-09-14

**Authors:** Sandra L. KLUND-HANSEN, Arve OPHEIM, Terje GJØVAAG, Eivind LUNDGAARD, Grethe MÅNUM, Linda RENNIE

**Affiliations:** 1 Department of Rehabilitation Science and Health Technology, Faculty of Health Sciences, OsloMet – Oslo Metropolitan University, Oslo; 2 Department of Rehabilitation, Technology and Innovation, Sunnaas Rehabilitation Hospital, Bjørnemyr; 3 Beitostølen Healthsports Center, Beitostølen; 4 Center for Habilitation and Rehabilitation Models and Services (CHARM), Faculty of Medicine, Institute of Health and Society, University of Oslo, Oslo, Norway

**Keywords:** adults, cerebral palsy, fatigue, follow-up study, pain, walking

## Abstract

**Objective:**

This study aimed to describe the long-term changes in walking function, fatigue, and pain in adults with cerebral palsy.

**Design:**

A 16-year follow-up study with paired comparisons.

**Subjects:**

Adults with spastic cerebral palsy (*n* = 29) from a baseline study in 2008.

**Methods:**

The mean age at follow-up was 50 (standard deviation 10) years.

**Primary outcomes:**

Gait Deviation Index, 6 Minute Walk Test, Timed-Up-and-Go test, walking speed, Fatigue Severity Scale, and bodily pain (visual analogue scale, 0–100). Paired samples *t*-test (Wilcoxon signed rank test for non-parametric data) was used to assess differences between baseline and follow-up. Between-group differences were analysed using an independent samples *t*-test (Mann–Whitney *U* test for non-parametric data).

**Results:**

Mean gait pattern deviations significantly (*p*-value = < 0.001) increased at follow-up compared with baseline for the full cohort. Walking speed decreased for the full cohort (–0.08 m/sec, *p*-value = 0.022), due to the bilateral group (–0.13 m/sec, *p*-value = 0.006). Walking capacity was maintained for the full cohort but decreased (mean diff: –84m, *p*-value = 0.035) for the bilateral group. Fatigue remained stable (*p*-value = 0.888). Pain decreased (*p*-value = 0.025) for the whole group, primarily due to the unilateral group (mean diff: 14 points on visual analogue scale, *p*-value = 0.031).

**Conclusions:**

Gait pattern deviations increased for adults with cerebral palsy during this 16-year follow-up. Walking speed and capacity decreased for the bilateral group but were maintained for the unilateral group. Fatigue symptoms were high at baseline but did not change across this follow-up. Pain decreased, similar to the general population.

## LAY ABSTRACT

This study investigated how walking, fatigue, and pain changed over 16 years in middle-aged adults with cerebral palsy. It involved 29 adults with spastic cerebral palsy who were first studied in 2008. By the time of follow-up, their average age was 50. The study measured walking patterns, speed, walking capacity, fatigue, and pain. Results showed that, overall, walking patterns got worse and walking speed slowed down, especially for those with bilateral cerebral palsy. While overall walking capacity remained the same, it dropped for those with bilateral cerebral palsy. Fatigue levels did not change over time. However, pain levels decreased, mainly for those with unilateral cerebral palsy, which aligns with trends seen in the general population. In conclusion, adults with cerebral palsy experience worsening walking difficulties as they age, bilateral more than unilateral. Although they have high fatigue levels, those levels stay stable, and they experience reduced pain over time.

Cerebral palsy (CP) is a complex condition arising from damage to the developing brain (1). It has traditionally been characterized as a childhood condition (2). Even though the brain damage causing the condition is stable, physical function is reported to decline during adulthood (3). In the last decades, there has been an increasing focus on adults with CP (4), which is now considered a lifespan disability (2).

Walking function normally deteriorates with age; however, this decline typically begins in late adulthood in healthy older adults (5). In contrast, adults with CP experience a different trajectory concerning walking function. Research shows that self-assessed walking function in individuals with CP declines significantly earlier, often as early as their mid-30s (6, 7). This premature decline can further affect the quality of life and independence (8). Additionally, increased pain (9) and fatigue (10) are commonly reported alongside declined self-perceived walking function (6). Interestingly, studies using objective measures of walking function, such as 3-dimensional gait analysis (3DGA), indicate no significant deterioration of gait patterns from adolescence to early adulthood (mean age at follow-up: 29 and 19, respectively) in individuals with CP (11, 12). Furthermore, MacCarthy et al. (13) found no association between gait pattern deviations (from 3DGA) and fatigue in a similarly aged cohort (median age 23).

Despite these insights, significant gaps remain in the literature concerning the longitudinal follow-up of middle-aged adults with CP. Most existing studies concentrate on childhood or early adulthood (14). Even though life expectancy is reported to be decreased compared with the general population, a large proportion of those with milder symptoms live well into older adulthood (15, 16). This leaves a critical period under-researched. Specifically, there is a lack of studies that utilize objective measures, such as 3DGA, to track changes in walking function alongside assessments of fatigue and pain over time in this population (4, 14). Addressing this knowledge gap is crucial for developing targeted interventions to maintain walking function, manage fatigue and pain, and thereby enhance the quality of life for middle-aged adults with CP.

Consequently, this study’s primary objective was to evaluate the long-term development of objective walking function measures. The secondary objective was to examine the development of fatigue and pain. The tertiary objective was to analyse possible differences in these measures between spastic unilateral cerebral palsy (SUCP) and spastic bilateral cerebral palsy (SBCP) subgroups.

## METHODS

### Study design

Baseline data were collected during a randomized controlled trial investigating the effect of Botulinum Toxin-A in ambulant adults with spastic CP on walking function and quality of life in 2008–2009 (17). The present study is a follow-up study with paired comparisons of the same participants at both measurement points. The follow-up data were collected in 2023–2024 with a mean of 16 years between measurement points.

### Ethical approval

The study was approved by the Regional Committee for Medical and Health Research Ethics (REK-number 530205) and data handling approval was obtained by the Norwegian Agency for Shared Services in Education and Research (SIKT-number 655023) before data collection.

### Participants

Inclusion criteria were participation in the baseline study of Månum et al. (17), spastic cerebral palsy, ability to walk 10 m with or without walking aids, and capacity to travel to a specialized hospital for data collection. The age limit was 65 at follow-up, due to expected change in gait parameters at ages over 65 in healthy older adults (18). Exclusion criteria were any ongoing health issues possibly affecting walking function reported by the participants and intellectual disabilities affecting the ability to supply informed consent. Treatment with Botulinumtoxin-A the last three months or surgery in lower limbs the last 1.5 years would exclude participation. Eligible participants provided written, informed consent and were invited for data collection at a specialized rehabilitation hospital.

### Data collection

*Demographic information.* Gross Motor Function Classification System (GMFCS) (19) level (self-reported, checked with other information on motor function from participants), relevant comorbidities, orthopedic surgery since baseline, physiotherapy service usage, exercise frequency and walking aids usage were collected (Table I).

**Table I:** Demographics and descriptive characteristics

Demographics	Baseline (2008)	Follow-up (2024)
Sex, *n*		
Men	14	14
Women	15	15
Age in years, mean (SD)	34 (10)	50 (10)
Type of cerebral palsy		
Unilateral	17	17
Bilateral	12	12
GMFCS level		
I	7	11
II	19	15
III	3	3
BMI, median (full range)	23 (19–39)	24 (19–45)
Marital status, *n*		
Single/Living alone	16	14
Married/cohabitant	12	13
Divorced/separated	1	1
Widow		1
Education, *n*		
First level (9 years)	3	2
Second level (12 years)	14	10
Third level (> 12 years)	12	17
Work/source of income, *n*		
Paid work (> 20%)	12	14
Student	6	1
Disablement benefit/unemployed	11	12
Retired	0	2
Physiotherapy, *n*		
Never/seldom	11	20
Every month	2	1
Every week	6	7
Several times a week	1	1
Exercise habits, *n*		
Never/seldom	7	7
Every month	2	1
Every week	6	6
Several times a week	14	15
Comorbidities, *n*		
Epilepsy		3
Allergy	3	4
Anxiety/depression		0
Hypertension	1	3
Hypothyroidism	2	1
Other	10	8
No comorbidities	16	13
Walking aids, *n*		
Orthopaedic shoes	7	5
Orthosis	3	13
Crutches	1	3
Walking frame	0	1
Manual wheelchair	3	2
Electric wheelchair	2	3
Orthopaedic surgery lower limb, *n*		Since 2008
Gastrocnemius/Soleus lengthening	17	4
Hamstrings lengthening	2	0
Iliopsoas lengthening	3	0
Ankle/foot	2	2
Other	4	0

Standard deviation: SD, *n*: number.

Height (cm) and bodyweight (kg) were measured upon arrival at the specialized hospital. Body mass index (BMI) was calculated with the following formula: weight(kg)/height(m^2^) (see Table I).

*Walking function.* The 6-Minute Walk Test (6MWT) measures the longest distance a person can walk in 6 min (20, 21). The participants walked back and forth along a 30-m corridor, turning around a cone at each end, wearing their usual shoes and using walking aids if necessary. The same test corridor was used at both baseline and follow-up. The 6MWT measures walking capacity and is a valid measure for persons with neurological diagnoses (21).

The Gait Deviation Index (GDI) (22) evaluates a person’s overall gait pattern deviations during walking, relative to a control group without gait impairments, using 3D gait analysis kinematics. The GDI is a valid and reliable measure of overall gait pattern deviations and treatment outcome assessment in adults and children (22–25).

The same motion analysis laboratory was used, with 6 Vicon Systems MX13 infrared cameras at baseline and 8 infrared Vantage cameras (Vicon Motion Systems, Oxford, UK) at follow-up. Ground reaction forces were measured synchronously with 2 embedded AMTI force plates (AMTI, Watertown, NY, USA). Retroreflective markers were placed on the pelvis and lower limbs according to the conventional gait model (Plug-in-gait, Vicon Motion Systems, Oxford, UK) and 3D data were processed using Vicon Nexus software (Vicon Motion Systems, Oxford, UK). Python (version 2.7; https://www.python.org/) was used to compute pelvis, hip, knee, and ankle kinematics and calculate GDI scores. At both baseline and follow-up participants walked barefoot at a self-selected, comfortable speed over a 10-m walkway until 3 trials with a minimum of 1 complete gait cycle, including valid kinetic data, were obtained.

Walking speed (m s^-1^) was measured during the 3D gait analysis and calculated from the Plug-in-gait model.

The Timed-Up-and-Go test (TUG) measures the time a person uses to stand up from a chair, walk 3 m, turn around, walk back, and sit down again (26). This is a measure of functional mobility by capturing the complex coordination of walking, balance, and general movements (27). The same test procedure and test location were used at baseline and follow-up.

*Fatigue.* The Fatigue Severity Scale (FSS) includes 9 statements that are rated on a Likert scale, ranging from 1 (strong disagreement) to 7 (strong agreement) according to the experiences during the last month (28). The Norwegian version of FSS was used (29). A mean fatigue score was used in the analysis. The FSS is validated for the general Norwegian population (29).

*Pain.* The bodily pain domain, from the RAND-36-Item Short Form Health Survey 1.0, reflects pain intensity and impact on daily life on a scale from 0 (worst possible pain) to 100 (no pain) (30, 31). The Norwegian version was used, and the Short Form Health Survey-36 (same questions as the RAND-36) shows good measurement properties for the general Norwegian population (32).

All questionnaires were unchanged from baseline to follow-up.

### Statistics

All statistical calculations were performed using IBM SPSS Statistics for Windows, version 29.0.2.0 (IBM Corp, Armonk, NY, USA). Descriptive data are presented as means and standard deviation (SD) for normally distributed variables, and medians and full range for ordinal or skewed variables.

The Shapiro–Wilk test was conducted to evaluate the normal distribution of the variables. For continuous, normally distributed data, the paired samples *t*-test was used to assess differences between baseline and follow-up. For variables violating the distribution of normality, the Wilcoxon signed rank test was conducted to evaluate differences between baseline and follow-up. Between-group differences were analyzed using an independent samples *t*-test for normally distributed data and Mann–Whitney *U* test for non-parametric data.

## RESULTS

### Inclusion

**Fig. 1:**
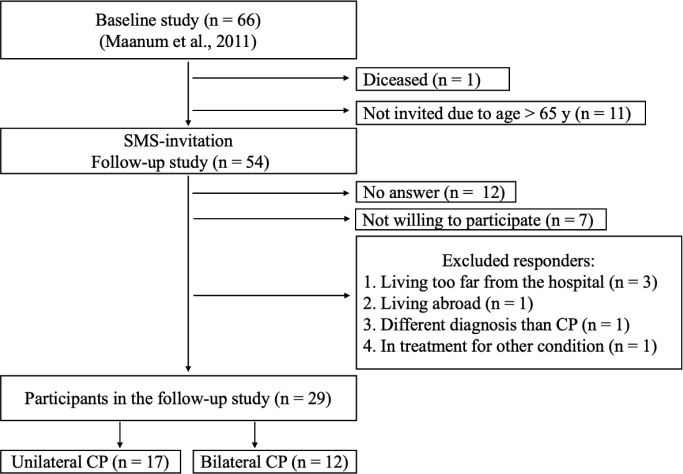
Flowchart of the inclusion process. CP: cerebral palsy.

Of the 66 participants in the original study (17), 1 was deceased at follow-up, and 11 were excluded due to age (> 65). Of the 54 deemed eligible for inclusion (see **Fig. 1** for flowchart of the inclusion process), 29 participants were recruited (see Table I for demographics and descriptive characteristics), with a response rate of 54%.

At follow-up, the self-reported GMFCS levels of 23 participants remained unchanged from baseline. One participant improved from level III to II, while another participant declined from II to III. Four participants progressed from GMFCS level II at baseline to level I at follow-up.

### Demographics and descriptive characteristics

**Fig. 2:**
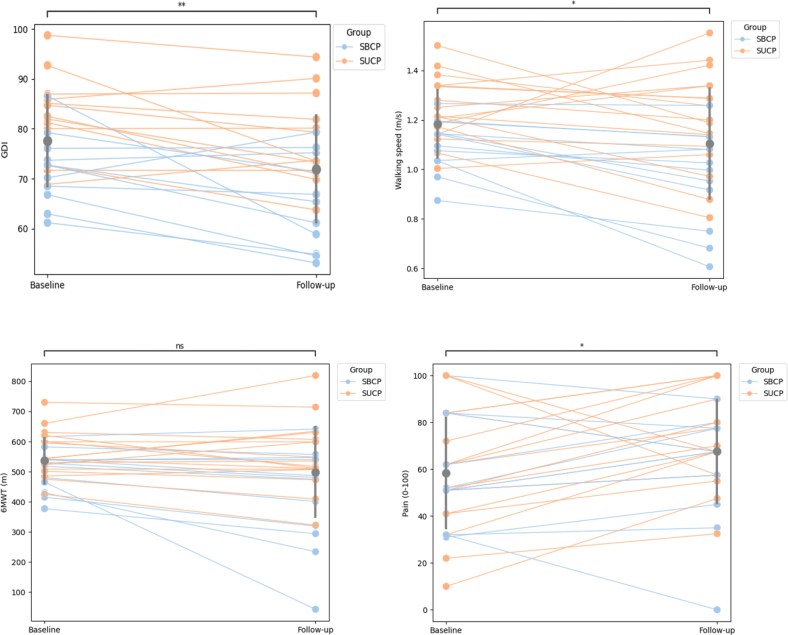
Spaghetti plot with individual lines for each participant and indication of SUCP (spastic unilateral cerebral palsy) and SBCP (spastic bilateral cerebral palsy) by colour, for GDI (Gait Deviation Index), 6MWT (6 Minute Walk Test), walking speed and pain (Pain Domain, RAND-36). Grey points with bars represent the group mean with 1 SD. Significance level of paired samples *t*-test: ns = not significant, * = significance level < 0.05, ** = significance level < 0.01.

**Fig. 3:**
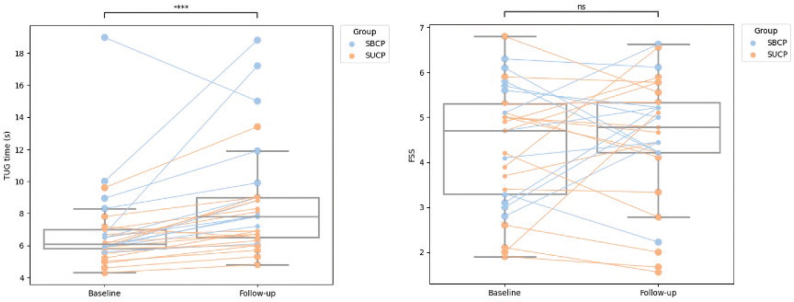
Boxplot, with median (IQR) changes in full cohort, with individual lines for each participant and indication of SUCP (spastic unilateral cerebral palsy) and SBCP (spastic bilateral cerebral palsy) by colour, for TUG (Timed-Up-and-Go test) and FSS (Fatigue Severity Scale). The box represents the interquartile range (IQR), with the horizontal line reflecting the median value. The whiskers are minimum to maximum values 1.,5 times IQR. Data points outside the whiskers are outliers. Significance level of the Wilcoxon signed-rank test: ns = not significant and **** = significance level < 0.0001.

*Walking function.* Gait pattern deviations increased significantly during the 16-year follow-up period (mean difference –5.6 points on GDI, 95% CI [2.5–8.8], *p* = < 0.001), and walking speed decreased significantly (mean difference: –0.08 m s^–1^, 95% CI [0.01–0.15], *p* = 0.022) for the whole group. Walking capacity (mean difference on 6MWT: –37 m, 95% CI [–1.62–76], *p* = 0.060) was, however, maintained for the whole group. Fig. 2 shows that the standard deviation in gait pattern deviations remains similar from baseline to follow-up, while the standard deviation in walking speed and capacity increases from baseline to follow-up. There are also 2 outliers with significantly reduced distances for walking capacity measurements. Functional mobility (TUG test) declined significantly for the entire group (z-score = 3.99, *p* = < 0.001) (Table II). For TUG times, the interquartile range (IQR) is larger at follow-up than at baseline, with prominent individual differences (Fig. 3).

**Table II:** Walking function, fatigue, and pain in participants with spastic cerebral palsy at baseline (2008) and at follow-up (2024)

Item	Baseline	Follow-up	*p*-value
GDI (0-100), mean (SD)			
All	77.6 (8.9)	72.0 (10.4)	< 0.001^*^
Bilateral	72.2 (7.0)	65.1 (8.9)	0.021^*^
Unilateral	81.6 (8.1)^a^	77.1 (8.2)	0.024^*^
Walking speed, (m s^-1^), mean (SD)			
All	1.18 (0.14)	1.10 (0.23)	0.022^*^
Bilateral	1.10 (0.11)	0.96 (0.20)	0.006^*^
Unilateral	1.25 (0.13)	1.20 (0.20)	0.369
6MWT (m), mean (SD)			
All	536 (78)	499 (153)	0.060
Bilateral	502 (71)	418 (168)	0.035^*^
Unilateral	560 (76)	556 (113)	0.824
TUG (s), median (IQR)			
All	6.1 (5.7–7.1)	7.8 (6.4–9.0)	< 0.001^*^
Bilateral	6.5 (5.9–8.8)	8.9 (7.8–14.2)	0.023^*^
Unilateral	5.9 (5.1–6.9)	6.7 (6.0–8.6)	< 0.001^*^
FSS (score 1–7), median (IQR)			
All	4.7 (3.2–5.5)	4.8 (4.2–5.4)	0.888
Bilateral	4.9 (3.2–5.8)	5.0 (4.3–5.2)	0.638
Unilateral	4.7 (3.0–5.1)	4.7 (3.1–5.7)	0.670
Bodily pain (0–100), mean (SD)			
All	58 (24)	68 (22)	0.025^*^
Bilateral	57 (22)	60 (24)	0.491
Unilateral	59 (26)	73 (20)	0.031^*^

* *p* < 0.05.

a The unilateral group had 1 missing. Test statistics for parametric data: mean, standard deviation (SD), and *p*-value from paired samples *t*-test. For non-parametric data: median value, interquartile range (IQR), and *p*-value from Wilcoxon signed rank test. GDI: Gait Deviation Index; 6MWT: Six Minute Walk Test; TUG: Timed-Up-and-Go test; FSS: Fatigue Severity Scale. bold letters and *p<0.05.

Between-group differences showed that those with SBCP had more gait pattern deviations (mean difference: 9.43, 95% CI [3.4–15.4], *p*-value: 0.003), lower walking speed (mean difference: 0.15 m s^–1^, 95% CI [0.05–0.24], *p*-value: 0.003), and lower walking capacity (mean difference: 57.2 m, 95% CI [0.1–114.2], *p*-value: 0.049) than the SUCP group at baseline.

Within-group analysis of SBCP from baseline to follow-up showed increased gait pattern deviations (mean difference = –7.1 points on GDI, 95% CI [1.3–13.0], *p* = 0.021), reduced walking speed (mean difference: –0.13, 95% CI [0.05–0.21], *p* = 0.006) and capacity (mean difference = – 84 m, 95% CI [7.4–161.5], *p* = 0.035), and decreased functional mobility (z-score = 2.28 on TUG, *p*-value = 0.023) (see Table II and Fig. 2). For the SUCP group, gait pattern deviations increased (mean difference = –4.5 points on GDI, 95% CI [0.7–8.4], *p* = 0.024), and functional mobility was reduced (z-score = 3.39, *p* = < 0.001) from baseline to follow-up. However, walking speed (mean difference = –0.05m s^–1^, 95% CI [–0.06–0.14], *p* = 0.369) and capacity (mean difference: –4 m, 95% CI [–33.6–41.6], *p* = 0.824) were maintained in this subgroup (see Table II and Fig. 2). Individual and group changes are presented in Figs 2 and 3.

*Fatigue and pain.* Fatigue scores for the whole group were unchanged from baseline to follow-up (z-score: –0.88, *p*-value: 0.381). There were no differences within subgroups (SBCP: z-scores: –0.47, *p*-value: 0.638 or SUCP: z-scores: –0.43, *p*-value: 0.670). However, the IQR is narrower at follow-up compared with baseline, and there are significant individual differences (see Fig. 3).

Reported pain decreased for the whole group (mean difference RAND-36 pain domain: 9.3, 95% CI [1.2–17.3], *p* = 0.025). For the SUCP group, pain decreased from baseline to follow-up (mean difference: 13.4, 95% CI [1.4–25.4], *p* = 0.031), while there were no changes in pain scores in the SBCP group (mean difference: 3.5, 95% CI [7.2–14.1], *p* = 0.491) (see Table II). Again, there are significant individual differences in these groups, which are presented in Fig. 2.

## DISCUSSION

### Development of walking function

The overall findings of this study revealed that adults with CP showed increased gait pattern deviations, with a further reduction in gait speed over 16 years into middle age. Walking capacity, however, remained unchanged. Interestingly, sub-group analysis revealed that the decrease in walking speed and walking capacity was only seen in the bilateral group, not in the unilateral group. Fatigue levels remained consistent over the 16 years for the whole group, while pain levels decreased for the unilateral group.

Our sample showed a greater change in gait pattern deviations (–5.6 points on the GDI) compared with previous reports in younger adults with CP (11–13). However, self-perceived walking function tends to decline in the mid-30s (6), suggesting that participants in earlier studies may not have experienced this decline yet. Our findings thereby support the results of Opheim et al. (6), indicating that changes in walking function occur in mid-life. Despite increasing gait pattern deviations and reduced walking speed over 16 years, the whole group did not decrease significantly (–37m, *p* = 0.060) in walking capacity (see Table II and Fig. 2).

It is relevant to note that the change in walking speed for the whole group (–0.08 m s^–1^) is not above the clinically relevant threshold of 0.1 m s^–1^ for individuals with pathologies (33). Further, gait pattern deviations increased for the whole group, exceeding 5 points on the GDI, which is considered clinically relevant for children with CP (22). However, no studies have determined the minimal clinically important difference or minimal detectable change for GDI in adults with CP. Research shows that the minimal detectable change is about 10 points for children with CP (24) and adults with stroke (34), raising questions regarding the validity of observed changes. This highlights the need for reliability studies to establish these metrics for adults with CP.

Further, the SBCP group increased their gait pattern deviations (–7.1, *p*-value: 0.021) more than the SUCP group (–4.5, *p* = 0.024*)*. This has previously been associated with increased flexion pattern and a shift in propulsion strategy from the ankle to the hip (35) in the bilateral group. Such a strategy may be more energy-demanding and add physical strain (5). Further, the SBCP group showed significantly decreased walking speed (–0.13 m s^–1^), which is considered clinically relevant (33), whereas the SUCP group maintained theirs. Lower walking speed is associated with increased ankle muscle stiffness (36, 37) and reduced force development in plantar flexors (37), which further correlates with self-reported physical function (38). Furthermore, the mean walking speed of the SBCP group was below 1.0 m s^–1^ at follow-up, indicating lower physical function and higher risk of functional limitations and need for care (39).

There were significant differences in 6MWT distance between CP subtypes at baseline (mean difference: 57m, *p* = 0.049) and follow-up (mean difference: 137m, *p* = 0.014). The SBCP group exhibited a significant reduction in 6MWT distance, with a mean difference of –84 m, where a change of 30–40 m is considered clinically relevant (40, 41), whereas the SUCP group did not deteriorate. Popliteal angle, pain, functional mobility (42), and plantar flexor strength (43) contribute to 6MWT performance in adults with CP. It is plausible that the decreasing walking function is associated with the above-mentioned functional limitations.

For most participants, GMFCS level remained stable, with improvements (*n* = 5) more often than deteriorations (*n* = 1). This contrasts with previous studies that typically report declines in self-perceived walking function in adults with CP (6, 44). However, Opheim et al. (6) also observed improvements in some participants, attributed to better muscle strength, balance, and fitness. This is supported by 21 of 29 participants who reported exercising at least once a week, with 15 exercising several times a week, likely contributing to the maintained gross motor function in this cohort. A recent review highlighted that combined aerobic and resistance training improved gross motor function, muscle strength, and gait speed more than usual care in persons with CP (45). However, there were only a limited number of studies including adults with CP in this review, underscoring the need for more research on adults with CP.

In addition, the whole group showed a decline in functional mobility. Although TUG test times increased significantly, the median time remained under 10 s, comparable to normative reference values, (46, 47), likely reflecting ageing by 16 years. Furthermore, of the 5 individuals who used more than 10 s on TUG at follow-up (see Fig. 3), 4 had SBCP. These 5 are the 3 GMFCS III participants and the 2 GMFCS II participants with the highest BMI and most pain symptoms. In addition, these 5 individuals walked the shortest distance on the 6MWT. This aligns with previous results where functional deterioration is associated with more severe neurological impairments, self-reported pain, and lack of physical activity (44).

### Development of fatigue and pain scores

Fatigue scores were high at baseline (median 4.7), compared with reported normative levels (mean 3.98) (29), and showed no significant changes at follow-up. This agrees with previous studies, reporting stable fatigue symptoms over time in adults with CP (6, 48). Fatigue has been associated with reduced self-perceived walking function (6). In contrast to previous research, fatigue scores remained unchanged despite considerable changes in walking function, especially for the SBCP group. This aligns with recent studies finding no significant correlations between objectively measured walking function and fatigue (13, 38), suggesting a discrepancy between objective and self-perceived walking function. Furthermore, these findings highlight the importance of early interventions to prevent chronic fatigue in adulthood for persons with CP. Notably, there are large individual differences in fatigue symptom development (see Fig. 3), reflecting the group’s heterogeneity. Previous studies have shown that higher age and reduced physical function were related to higher levels of fatigue symptoms (49). This highlights that this study is generalizable only to the study population, middle-aged walking adults with CP.

Bodily pain decreased in the whole group from baseline to follow-up, with a mean difference of –9.3 points on the RAND36 bodily pain score. The change is primarily due to the unilateral group changing from a mean score of 59 at baseline to 73 at follow-up, comparable to the mean pain score of 72 for ages 40–60 in the normative Norwegian population (32). This reduction may be due to age, as lower pain prevalence is reported in older adults compared with those aged 30–35 years (50), or possibly because individuals with more pain opted out of participation.

Previous studies have shown that exercise alleviates pain (51, 52), while inactivity is associated with increased pain (53). In this study, two-thirds of participants exercised at least once a week, which may have positively influenced their pain experience. Conversely, pain did not decrease for the SBCP group, possibly due to an overall health decline, as indicated by reduced walking speed, walking capacity, and greater gait pattern deviations. Lower functional ability has, in some studies, been shown to relate to more pain (6, 9). Furthermore, the overall change in pain in this sample may have been influenced by the fact that this study included only walking participants, which reflects higher functional ability within the CP population.

### Methodological considerations

A considerable number of non-responders might have affected the results. In the baseline study (17), there were 36/66 with SBCP, compared with 12/29 with SBCP at follow-up. For GMFCS levels, 7 out of 9 on level I, 19 of 48 on level II, and 3 out of 9 on level III from the baseline study are included. In addition, baseline participants aged above 65 were excluded (*n* = 11). As previously mentioned, this suggests that more individuals with higher physical function were included in the follow-up, making the results more generalizable to a higher-functioning CP population. However, as this is a comparative study, with the same sample at baseline and follow-up, the impact of the non-responders is minimized.

The small sample size, especially in subgroup analyses, warrants cautious interpretation. Participation in the baseline study (17) may have influenced the sample, as inclusion criteria required experiencing a reduction in walking function, potentially affecting the changes observed.

In conclusion, this long-term follow-up study is particularly significant as it fills a gap in knowledge of how objectively measured walking function evolves in adults with CP over a 16-year follow-up period.

Middle-aged adults with CP experience increased gait pattern deviations and lower walking speed during adulthood, individuals with SBCP being more severely affected than those with SUCP. Fatigue symptoms are notably high, yet consistent over 16 years.

The SBCP group had deterioration of all walking function measures: walking speed, capacity, gait pattern deviations, and functional mobility, and maintained pain symptoms. The SUCP group maintained their walking speed and capacity, while pain symptoms decreased.

Although functional mobility tends to decline in adults with CP, it does so within the expected change with ageing. This underscores the resilience and adaptability of adults with CP navigating the complex challenges of ageing with this condition.

## References

[1] Rosenbaum P, Paneth N, Leviton A, Goldstein M, Bax M, Damiano D, et al. A report: the definition and classification of cerebral palsy April 2006. Dev Med Child Neurol Suppl 2007; 109: 8–14. 17370477

[2] Morgan P, McGinley JL. Cerebral palsy. Handbook of Clinical Neurology 2018; 159: 323–336. 10.1016/B978-0-444-63916-5.00020-3 30482324

[3] Jonsson U, Eek MN, Sunnerhagen KS, Himmelmann K. Changes in walking ability, intellectual disability, and epilepsy in adults with cerebral palsy over 50 years: a population-based follow-up study. Dev Med Child Neurol 2021; 63: 839–845. 10.1111/dmcn.14871 33772773

[4] Benner JL, Noten S, Limsakul C, Van Der Slot WMA, Stam HJ, Selb M, et al. Outcomes in adults with cerebral palsy: systematic review using the International Classification of Functioning, Disability and Health. Dev Med Child Neurol 2019; 61: 1153–1161. 10.1111/dmcn.14247 30985004

[5] Boyer KA, Hayes KL, Umberger BR, Adamczyk PG, Bean JF, Brach JS, et al. Age-related changes in gait biomechanics and their impact on the metabolic cost of walking: Report from a National Institute on Aging workshop. Exp Gerontol 2023; 173: 112102. 10.1016/j.exger.2023.112102 36693530 PMC10008437

[6] Opheim A, Jahnsen R, Olsson E, Stanghelle JK. Walking function, pain, and fatigue in adults with cerebral palsy: a 7-year follow-up study. Dev Med Child Neurol 2009; 51: 381–388. 10.1111/j.1469-8749.2008.03250.x 19207296

[7] Benner JL, Hilberink SR, Veenis T, Stam HJ, van der Slot WM, Roebroeck ME. Long-term deterioration of perceived health and functioning in adults with cerebral palsy. Arch Phys Med Rehabil 2017; 98: 2196–2205. 10.1016/j.apmr.2017.03.013 28427924

[8] Ostensjø S, Carlberg EB, Vøllestad NK. Motor impairments in young children with cerebral palsy: relationship to gross motor function and everyday activities. Dev Med Child Neurol 2004; 46: 580–589. 10.1111/j.1469-8749.2004.tb01021.x 15344517

[9] van der Slot WMA, Benner JL, Brunton L, Engel JM, Gallien P, Hilberink SR, et al. Pain in adults with cerebral palsy: a systematic review and meta-analysis of individual participant data. Ann Phys Rehabil Med 2021; 64: 101359. 10.1016/j.rehab.2019.12.011 32061920

[10] Puce L, Pallecchi I, Chamari K, Marinelli L, Innocenti T, Pedrini R, et al. Systematic review of fatigue in individuals with cerebral palsy. Front Hum Neurosci 2021; 15: 598800. 10.3389/fnhum.2021.598800 33790748 PMC8005578

[11] Lennon N, Church C, Shrader MW, Robinson W, Henley J, Salazar-Torres JJ, et al. Mobility and gait in adults with cerebral palsy: evaluating change from adolescence. Gait Posture 2021; 90: 374–379. 10.1016/j.gaitpost.2021.09.177 34564009

[12] Bonnefoy-Mazure A, De Coulon G, Lascombes P, Bregou A, Armand S. A 10.5-year follow-up of walking with unilateral spastic cerebral palsy. J Child Orthop 2023; 17: 173–183. 10.1177/18632521231154975 37034199 PMC10080234

[13] MacCarthy M, Heyn P, Tagawa A, Carollo J. Walking speed and patient-reported outcomes in young adults with cerebral palsy. Dev Med Child Neurol 2022; 64: 1281–1288. 10.1111/dmcn.15225 35366333

[14] Gravholt A, Fernandez B, Bessaguet H, Millet GY, Buizer AI, Lapole T. Motor function and gait decline in individuals with cerebral palsy during adulthood: a narrative review of potential physiological determinants. Eur J Appl Physiol 2024; 124: 2867–2879. 10.1007/s00421-024-05550-y 39042142

[15] Blair E, Langdon K, McIntyre S, Lawrence D, Watson L. Survival and mortality in cerebral palsy: observations to the sixth decade from a data linkage study of a total population register and National Death Index. BMC Neurol 2019; 19: 111. 10.1186/s12883-019-1343-1 31164086 PMC6549269

[16] Himmelmann K, Sundh V. Survival with cerebral palsy over five decades in western Sweden. Dev Med Child Neurol 2015; 57: 762–767. 10.1111/dmcn.12718 25694102

[17] Maanum G, Jahnsen R, Stanghelle JK, Sandvik L, Keller A. Effects of botulinum toxin A in ambulant adults with spastic cerebral palsy: a randomized double-blind placebo controlled-trial. J Rehabil Med 2011; 43: 338–347. 10.2340/16501977-0672 21305227

[18] Schrack JA, Zipunnikov V, Simonsick EM, Studenski S, Ferrucci L. Rising energetic cost of walking predicts gait speed decline with aging. J Gerontol A Biol Sci Med Sci 2016; 71: 947–953. 10.1093/gerona/glw002 26850913 PMC4906328

[19] McCormick A, Brien M, Plourde J, Wood E, Rosenbaum P, McLean J. Stability of the Gross Motor Function Classification System in adults with cerebral palsy. Dev Med Child Neurol 2007; 49: 265–269. 10.1111/j.1469-8749.2007.00265.x 17376136

[20] Guyatt GH, Sullivan MJ, Thompson PJ, Fallen EL, Pugsley SO, Taylor DW, et al. The 6-minute walk: a new measure of exercise capacity in patients with chronic heart failure. Can Med Assoc J 1985; 132: 919–923. 3978515 PMC1345899

[21] ATS statement: guidelines for the six-minute walk test. Am J Resp Crit Care Med 2002; 166: 111–117. 10.1164/ajrccm.166.1.at1102 12091180

[22] Schwartz MH, Rozumalski A. The Gait Deviation Index: a new comprehensive index of gait pathology. Gait Posture 2008; 28: 351–357. 10.1016/j.gaitpost.2008.05.001 18565753

[23] Maanum G, Jahnsen R, Stanghelle JK, Sandvik L, Larsen KL, Keller A. Face and construct validity of the Gait Deviation Index in adults with spastic cerebral palsy. J Rehabil Med 2012; 44: 272–275. 10.2340/16501977-0930 22214985

[24] Rasmussen HM, Nielsen DB, Pedersen NW, Overgaard S, Holsgaard-Larsen A. Gait Deviation Index, Gait Profile Score and Gait Variable Score in children with spastic cerebral palsy: intra-rater reliability and agreement across two repeated sessions. Gait Posture 2015; 42: 133–137. 10.1016/j.gaitpost.2015.04.019 26043670

[25] McMulkin ML, MacWilliams BA. Application of the Gillette Gait Index, Gait Deviation Index and Gait Profile Score to multiple clinical pediatric populations. Gait Posture 2015; 41: 608–612. 10.1016/j.gaitpost.2015.01.005 25623856

[26] Mathias S, Nayak US, Isaacs B. Balance in elderly patients: the “get-up and go” test. Arch Phys Med Rehabil, 1986; 67: 387–389. 3487300

[27] Podsiadlo D, Richardson S. The timed “Up & Go”: a test of basic functional mobility for frail elderly persons. J Am Geriatr Soc 1991; 39: 142–148. 10.1111/j.1532-5415.1991.tb01616.x 1991946

[28] Krupp LB, LaRocca NG, Muir-Nash J, Steinberg AD. The Fatigue Severity Scale: application to patients with multiple sclerosis and systemic lupus erythematosus. Arch Neurol 1989; 46: 1121–1123. 10.1001/archneur.1989.00520460115022 2803071

[29] Lerdal A, Wahl A, Rustoen T, Hanestad BR, Moum T. Fatigue in the general population: a translation and test of the psychometric properties of the Norwegian version of the fatigue severity scale. Scand J Public Health 2005; 33: 123–130. 10.1080/14034940410028406 15823973

[30] Ware JE Jr, Sherbourne CD. The MOS 36-item short-form health survey (SF-36). I. Conceptual framework and item selection. Med Care 1992; 30: 473–483. 10.1097/00005650-199206000-00002 1593914

[31] Hays RD, Sherbourne CD, Mazel RM. The RAND 36-Item Health Survey 1.0. Health Econ 1993; 2: 217–227. 10.1002/hec.4730020305 8275167

[32] Garratt AM, Stavem K. Measurement properties and normative data for the Norwegian SF-36: results from a general population survey. Health Qual Life Outcomes 2017; 15: 51. 10.1186/s12955-017-0625-9 28292292 PMC5351285

[33] Bohannon RW, Glenney SS. Minimal clinically important difference for change in comfortable gait speed of adults with pathology: a systematic review. J Eval Clin Pract 2014; 20: 295–300. 10.1111/jep.12158 24798823

[34] Correa KP, Devetak GF, Martello SK, de Almeida JC, Pauleto AC, Manffra EF. Reliability and Minimum Detectable Change of the Gait Deviation Index (GDI) in post-stroke patients. Gait Posture 2017; 53: 29–34. 10.1016/j.gaitpost.2016.12.012 28073084

[35] Lundh S, Nasic S, Riad J. Fatigue, quality of life and walking ability in adults with cerebral palsy. Gait Posture 2018; 61: 1–6. 10.1016/j.gaitpost.2017.12.017 29277025

[36] Svane C, Forman CR, Rasul A, Nielsen JB, Lorentzen J. Muscle contractures in adults with cerebral palsy characterized by combined ultrasound-derived echo intensity and handheld dynamometry measures. Ultrasound Med Biol 2022; 48: 694–701. 10.1016/j.ultrasmedbio.2021.12.012 35065812

[37] Geertsen SS, Kirk H, Lorentzen J, Jorsal M, Johansson CB, Nielsen JB. Impaired gait function in adults with cerebral palsy is associated with reduced rapid force generation and increased passive stiffness. Clin Neurophysiol 2015; 126: 2320–2329. 10.1016/j.clinph.2015.02.005 25757398

[38] Baer HR, Thomas SP, Pan Z, Tagawa A, Carollo JJ, Heyn PC. Self-reported physical function is associated with walking speed in adults with cerebral palsy. J Pediatr Rehabil Med 2019; 12: 181–188. 10.3233/PRM-180585 31227669

[39] Middleton A, Fritz SL, Lusardi M. Walking speed: the functional vital sign. J Aging Phys Act 2015; 23: 314–322. 10.1123/japa.2013-0236 24812254 PMC4254896

[40] Bohannon RW, Crouch R. Minimal clinically important difference for change in 6-minute walk test distance of adults with pathology: a systematic review. J Eval Clin Pract 2017; 23: 377–381. 10.1111/jep.12629 27592691

[41] Igarashi T, Miyata K, Tamura S, Otani T, Iizuka T, Usuda S. Minimal clinically important difference in 6-minute walk distance estimated by multiple methods in inpatients with subacute cardiovascular disease. Physiother Theory Pract 2024; 40: 1981–1989. 10.1080/09593985.2023.2232014 37395670

[42] Maanum G, Jahnsen R, Frøslie KF, Larsen KL, Keller A. Walking ability and predictors of performance on the 6-minute walk test in adults with spastic cerebral palsy. Dev Med Child Neurol 2010; 52: e126–132. 10.1111/j.1469-8749.2010.03614.x 20163429

[43] Gillett JG, Lichtwark GA, Boyd RN, Barber LA. Functional capacity in adults with cerebral palsy: lower limb muscle strength matters. Arch Phys Med Rehabil 2018; 99: 900–6.e1. 10.1016/j.apmr.2018.01.020 29438658

[44] Jahnsen R, Villien L, Egeland T, Stanghelle JK, Holm I. Locomotion skills in adults with cerebral palsy. Clin Rehabil 2004; 18: 309–316. 10.1191/0269215504cr735oa 15137562

[45] Wang B, Huang H. Effects of various exercise interventions on motor function in cerebral palsy patients: a systematic review and network meta-analysis. Neurol Sci 2024; 45: 5915–5927. 10.1007/s10072-024-07741-z 39190170

[46] Kear BM, Guck TP, McGaha AL. Timed Up and Go (TUG) Test: normative reference values for ages 20 to 59 years and relationships with physical and mental health risk factors. J Prim Care Community Health 2017; 8: 9–13. 10.1177/2150131916659282 27450179 PMC5932649

[47] Bohannon RW. Reference values for the timed up and go test: a descriptive meta-analysis. J Geriatr Phys Ther 2006; 29: 64–68. 10.1519/00139143-200608000-00004 16914068

[48] Oude Lansink ILB, McPhee PG, Brunton LK, Gorter JW. Fatigue in adults with cerebral palsy: a three-year follow-up study. J Rehabil Med 2018; 50: 886–891. 10.2340/16501977-2493 30299521

[49] Jahnsen R, Villien L, Stanghelle JK, Holm I. Fatigue in adults with cerebral palsy in Norway compared with the general population. Dev Med Child Neurol 2003; 45: 296–303. 10.1111/j.1469-8749.2003.tb00399.x 12729142

[50] Rodby-Bousquet E, Alriksson-Schmidt A, Jarl J. Prevalence of pain and interference with daily activities and sleep in adults with cerebral palsy. Dev Med Child Neurol 2021; 63: 60–67. 10.1111/dmcn.14678 32951227 PMC7756851

[51] Engel JM, Kartin D, Jensen MP. Pain treatment in persons with cerebral palsy: frequency and helpfulness. Am J Phys Med Rehabil/Assoc Academic Physiatrists 2002; 81: 291–296. 10.1097/00002060-200204000-00009 11953547

[52] Hirsh AT, Kratz AL, Engel JM, Jensen MP. Survey results of pain treatments in adults with cerebral palsy. Am J Phys Med Rehabil/Assoc Academic Physiatrists 2011; 90: 207–216. 10.1097/PHM.0b013e3182063bc9 PMC3036542 21273894

[53] Jahnsen R, Villien L, Aamodt G, Stanghelle JK, Holm I. Musculoskeletal pain in adults with cerebral palsy compared with the general population. J Rehabil Med 2004; 36: 78–84. 10.1080/16501970310018305 15180222

